# Machine Learning-Based Model for Prediction of Hemorrhage Transformation in Acute Ischemic Stroke After Alteplase

**DOI:** 10.3389/fneur.2022.897903

**Published:** 2022-06-10

**Authors:** Yanan Xu, Xiaoli Li, Di Wu, Zhengsheng Zhang, Aizhong Jiang

**Affiliations:** ^1^Department of Neurology, ZhongDa Hospital Southeast University (JiangBei) (NanJing DaChang Hospital), Nanjing, China; ^2^Department of Neurology, Affiliated ZhongDa Hospital, Southeast University, Nanjing, China

**Keywords:** stroke, hemorrhage, machine learning, random forest, prediction

## Abstract

Hemorrhage transformation (HT) is the most dreaded complication of intravenous thrombolysis (IVT) in acute ischemic stroke (AIS). The prediction of HT after IVT is important in the treatment decision-making for AIS. We designed and compared different machine learning methods, capable of predicting HT in AIS after IVT. A total of 345 AIS patients who received intravenous alteplase between January 2016 and June 2021 were enrolled in this retrospective study. The demographic characteristics, clinical condition, biochemical data, and neuroimaging variables were included for analysis. HT was confirmed by head computed tomography (CT) or magnetic resonance imaging (MRI) within 48 h after IVT. Based on the neuroimaging results, all of the patients were divided into the non-HT group and the HT group. Then, the variables were applied in logistic regression (LR) and random forest (RF) algorithms to establish HT prediction models. To evaluate the accuracy of the machine learning models, the models were compared to several of the common scales used in clinics, including the multicenter stroke survey (MSS) score, safe implementation of treatments in stroke (SITS) score, and SEDAN score. The performance of these prediction models was evaluated using the receiver operating characteristic (ROC) curve (AUC). Forty-five patients had HT (13.0%) within 48 h after IVT. The ROC curve results showed that the AUCs of HT that were predicted by the RF model, LR model, MSS, SITS, and SEDAN scales after IVT were 0.795 (95% CI, 0.647–0.944), 0.703 (95% CI, 0.515–0.892), 0.657 (95% CI, 0.574–0.741), 0.660 (95% CI, 0.580–0.740) and 0.655 (95% CI, 0.571–0.739), respectively. The RF model performed better than the other models and scales. The top four most influential factors in the RF importance matrix plot were triglyceride, Lpa, the baseline NIHSS, and hemoglobin. The SHapley Additive exPlanation values made the RF prediction model clinically interpretable. In this study, an RF machine learning method was successfully established to predict HT in AIS patients after intravenous alteplase, which the sensitivity was 66.7%, and the specificity was 80.7%.

## Introduction

Stroke is a major global public health issue with high morbidity and mortality. According to China's Ministry of Health, 77.8% of strokes are ischemic (1). Currently, poststroke outcomes are significantly improved by reperfusion therapy, such as intravenous thrombolysis and endovascular thrombectomy (2–6). Despite its efficacy, IVT also increases the risk of the development of hemorrage transformation (HT). The occurrence of HT significantly affects functional recovery and is independently associated with a higher mortality (7). It was shown in previous studies that a total of 10–48% of the AIS patients who received IVT developed HT (8) and the incidence of symptomatic intracranial hemorrhage (SICH) ranged from 2 to 7% (9). There is currently a lack of understanding of the benefits of IVT relative to its risks, which include HT, in both clinicians and patients (or their relatives). This results in patients not always receiving a timely intervention.

Previous reports have indicated that there are a number of clinical, laboratory, and radiographic factors associated with the risk of HT after alteplase. A systematic review and meta-analysis of 55 studies have reported on factors that are associated with HT, and these included age; the severity of the stroke; the baseline glucose; the use of antiplatelet drugs or statins; leukoaraiosis; early signs of infarction on head CT; the presence of atrial fibrillation, diabetes, previous ischemic heart disease or cerebral vascular diseases; and congestive heart failure (10). Lipoprotein (11), the neutrophil-to-lymphocyte ratio (NLR) (12) and the serum homocysteine level (13) were also reported as factors associated with HT after IVT. Numerous scoring systems have been devised to assess the risk for HT in AIS patients after IVT, and these include the Hemorrhage After Thrombolysis (HAT) score (14), multicenter stroke survey (MSS) score (15), safe implementation of treatments in stroke (SITS) score (16), SEDAN score (17) and GRASPS score (18). However, existing HT models cannot address the full complexity of all the factors that are involved. Each of the methods has limitations and disadvantages (14–18), which implies a certain degree of inaccuracy. The risk of HT after IVT in AIS patients cannot be avoided to the greatest extent when the stroke patients are only assessed based on the existing assessment system. Therefore, it is an urgent priority to find more reliable and effective methods to develop an early and timely prediction for HT after IVT in AIS patients.

Machine learning (ML) is a branch of artificial intelligence that can establish ideal models for classification, prediction, and estimation by allowing computers to “learn” from large, noisy or complex input and output datasets (19). In recent years, machine learning has been applied in medical research. For example, machine learning has been applied for the prediction of the outcomes in acute stroke patients (20) and endovascular treatment outcome in AIS (21). In the present study, we developed machine learning-based models that included a logistic regression (LR) model and a random forest (RF) model to predict HT. We then compared the predictability to several scales that are currently used in the clinic, including the MSS score, SITS score and SEDAN score.

## Materials and Methods

### Participants

AIS patients treated with rt-PA therapy after admission to Zhongda Hospital Affiliated to Southeast University between 1 January 2016 and 30 June 2021 were enrolled in this retrospective study. Inclusion criteria were as follows: (1) age ≥ 18 years, (2) AIS confirmed by magnetic resonance imaging (MRI), and (3) onset of stroke symptoms within 4.5 h and treated with rt-PA. Exclusion criteria were as follows: (1) additional endovascular therapy after intravenous thrombolysis, (2) no head computed tomography (CT) or MRI patients within 48 h after intravenous thrombolysis, (3) patients treated with urokinase thrombolytic therapy. This retrospective study was approved by the Research Ethics Committee of Affiliated ZhongDa Hospital and the Southeast University (approval number: 2021ZDSYLL300-P02).

### Data Collection

The clinical data were collected by a certified stroke neurologist. Overall, 61 clinical variables were initially selected for the model construction.

The study collected demographic data including patient sex, age, body weight, past history (including hypertension, diabetes, coronary artery disease, atrial fibrillation, stroke, antiplatelet therapy, smoking, and alcohol consumption), clinical characteristics, including baseline indicators (the baseline NIHSS, the baseline blood pressure, early infarction signs seen on a head CT on admission and the baseline laboratory tests), the time from onset to thrombolytic therapy (OTT), the rt-PA dose (0.9 or 0.6 mg/kg), the NIHSS score (1 h after IVT), the NIHSS score (2 h after IVT), and the laboratory test results on the second day after IVT.

### HT Scores

The SEDAN, MSS, and SITS scores were used for the measuring the HT scores, all of the patients were evaluated based on the scale, and they were evaluated after IVT and after having medications for 48 h. When the score was higher, there was a greater risk that the AIS patient would develop HT after IVT.

### Machine-Learning Algorithms

To compare the machine-learning risk algorithms, the study population was split into a “training” cohort from which the HT risk algorithms were derived and a “validation” cohort in which the algorithms were applied and tested. The “training” cohort was derived from a random sampling of 80% of the AIS patients, and the “validation” cohort was comprised of the remaining 20% of the AIS patients. The machine learning models were trained with all of the variables as inputs to classify the patients who were likely to have HT after IVT for AIS. Two of the commonly used classes of machine-learning algorithms were utilized: logistic regression, and random forest. To evaluate the accuracy of the machine learning models, we calculated three of the common scales used in the clinic as references: the MSS score, SITS score and SEDAN score. An ROC (receiver operating characteristic) curve was used to evaluate the performance of the models.

The effects of the features on the RF prediction model were measured using the functions of the SHapley Additive exPlanations (SHAP) Python package (version 0.40.0), which assessed the importance of each feature using a game-theoretic approach (22).

### Statistical Analysis

SPSS 22.0 was used for the descriptive analysis and the comparison of clinically defined groups. Continuous variables are expressed as means ± SD or as medians (interquartile range). Categorical variables are expressed as percentages. Continuous data were compared between the groups using *t*-test or a non-parametric test. Categorical data were compared using the χ2 test. *P*-values were corrected for multiple testing with the Benjamini-Hochberg false discovery rate (FDR) correction using the Statsmodels package in Python. In all cases, FDR-adjusted *P* < 0.05 was considered to indicate statistical significance.

## Results

**Figure 1:**
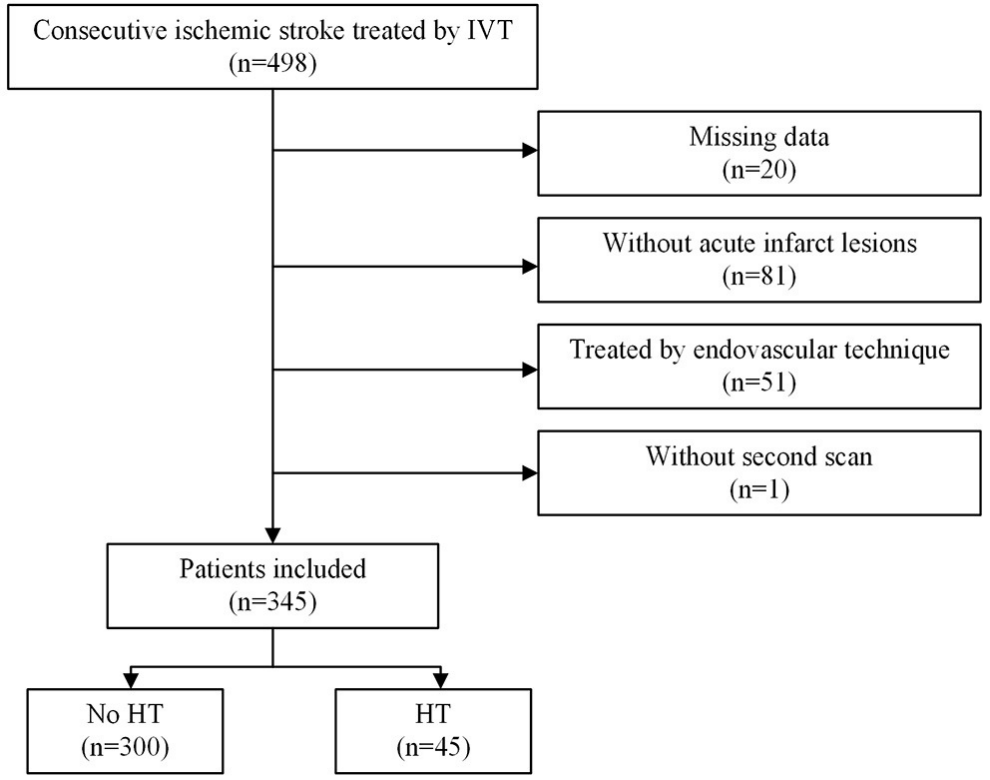
Flow chart illustrating patient selection. IVT indicates introvenous thrombolysis. HT indicates hemorrhage transformation. IVT, intravenous thrombolysis; HT, hemorrhage transformation.

**Figure 2:**
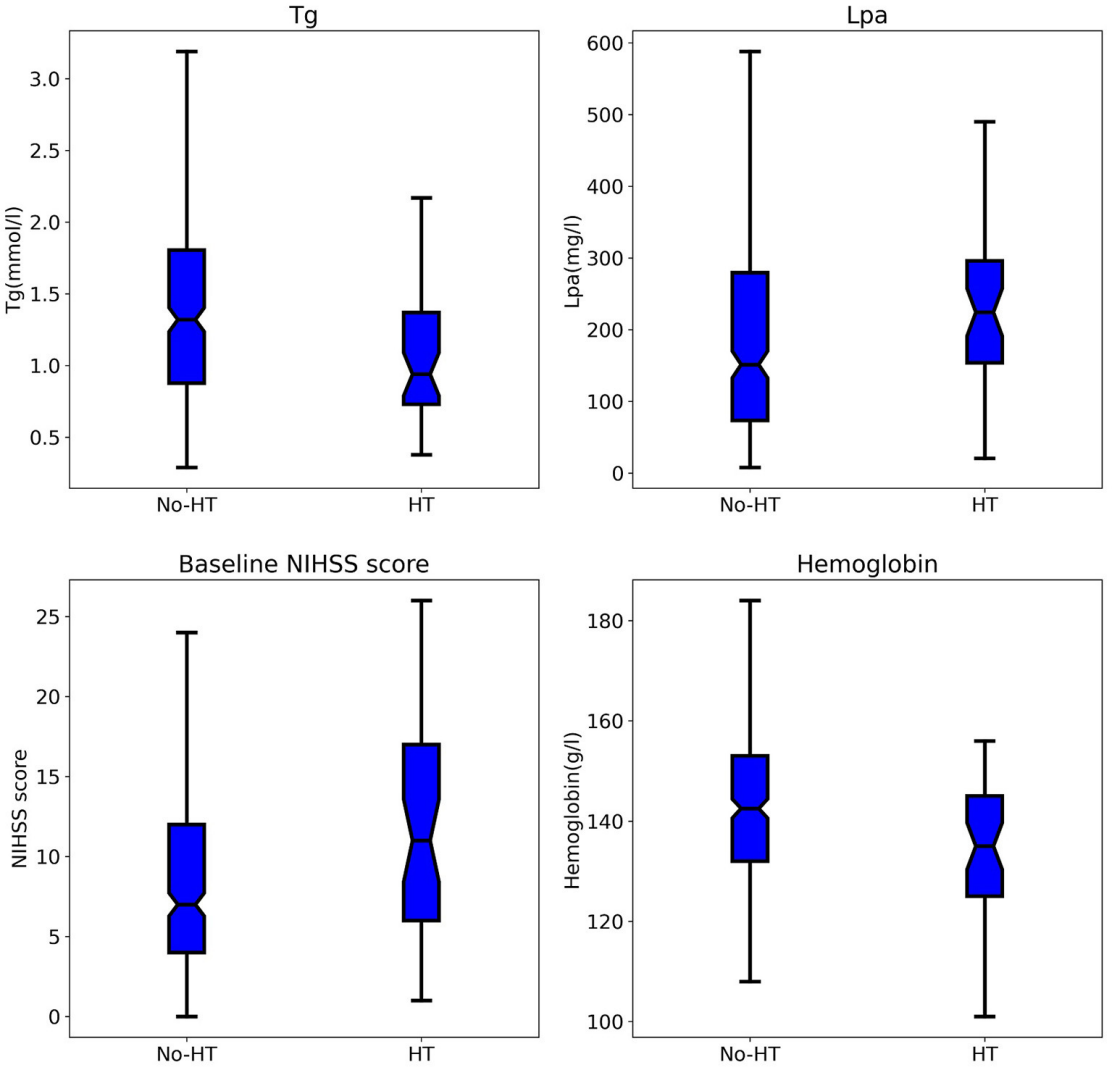
Comparison of Tg, Lpa, baseline NIHSS score, hemoglobin between patients in the HT and no-HT group. Tg, triglycerides; Lpa, lipoprotein(a); HT, hemorrhage transformation.

A total of 498 patients who received IVT with rt-PA within 4.5 h of stroke onset were included in the cohort during the study period. Ultimately, 345 patients were included (Figure 1), after excluding 20 patients with missing laboratory tests or clinical data, 81 patients who did not have acute infarct lesions that were found on DWI, 51 patients who underwent endovascular therapy, and 1 patient without a second scan. The study sample included 121 (35.1%) females and 224 (64.9%) males. Their ages ranged from 29 to 95 years with a median (IQR) of 70 years (63–81). Forty-five (13.0%) patients developed HT, and ten (2.9%) patients developed SICH (defined as a clinical deterioration expressed by an increase of at least 4 points on the NIHSS scale). The patients who developed HT were older (FDR *p*-value: 0.026), had higher baseline NIHSS scores (FDR *p*-value: 0.009), NIHSS scores 1 h after IVT (FDR *p*-value: 0.009), NIHSS scores 2 h after IVT (FDR *p*-value: 0.014), PT values (FDR *p*-value: 0.014), and INR values (FDR *p*-value: 0.043), and they had lower RBC counts (FDR *p*-value: 0.009), hemoglobin (FDR *p*-value: 0.009), lymphocytes (FDR *p*-value: 0.029), and triglycerides after IVT (FDR *p*-value: 0.009). Also, more of patients who developed HT had cardioembolic events (FDR *p*-value: 0.044) compared to the non-HT group. However, there were less HT patients that had small vessel occlusion (FDR *p*-value: 0.009) compared to the non-HT group. The demographic and clinical characteristics of this study population are shown in Table 1, Figure 2.

**Table 1:** Demographic and clinical characteristics of the study sample.

**Parameter**	**HT** **(*n* = 45)**	**No-HT** **(*n* = 300)**	**t/χ2/z** **value**	***P-*value**	**FDR-adjusted *P-*value**
Age, years	78 (68, 82)	70 (63, 80)	−2.847	0.004	0.026
Body weight, kg	60 (54, 70)	66 (55, 72)	−1.424	0.154	0.282
**Sex**					
Male	24 (53.3%)	200 (66.7%)	3.055	0.080	0.190
**Past history**					
Smoking	10 (22.2%)	115 (38.3%)	4.396	0.036	0.121
Alcohol consumption	5 (11.1%)	62 (20.7%)	2.283	0.131	0.273
**Comorbidities**					
Hypertension, *n* (%)	29 (64.4%)	210 (70.0%)	0.567	0.451	0.656
Diabetes, *n* (%)	10 (22.2%)	81 (27.0%)	0.460	0.498	0.694
Coronary artery disease, *n* (%)	7 (15.6%)	39 (13.0%)	0.221	0.638	0.772
Atrial fibrillation, *n* (%)	15 (33.3%)	58 (19.3%)	4.597	0.032	0.114
Previous stroke, *n* (%)	13 (28.9%)	75 (25.0%)	0.311	0.577	0.754
PAT	6 (13.3%)	34 (11.3%)	0.153	0.696	0.810
Statin use	3 (6.7%)	13 (4.3%)	0.099	0.754	0.815
OTT	180 (120, 238)	165 (120, 200)	−1.147	0.252	0.428
SBP on admission	159 (148, 175)	152 (139, 164)	−2.302	0.021	0.084
DBP on admission	84.9 ± 14.1	85.5 ± 14.4	0.267	0.789	0.828
Rt-pa dose			0.457	0.499	0.694
0.9 mg/kg	33 (73.3%)	205 (68.3%)			
0.6 mg/kg	22 (26.7%)	95 (31.7%)			
Early infarction signs on admission with head CT	5 (11.1%)	25 (8.3%)	0.111	0.739	0.815
Circulation, *n* (%)			4.441	0.109	0.241
Anterior circulation infarct	38 (84.4%)	236 (78.7%)			
Posterior circulation infarct	3 (6.7%)	50 (16.7%)			
Both	4 (8.9%)	14 (4.7%)			
Leukoaraiosis	35 (77.8%)	201 (67.0%)	2.103	0.147	0.277
Stroke subtype, *n* (%)			14.185	0.001	0.009
Cardioembolic	13 (28.9%)	41 (13.7%)	6.868	0.009	0.044
Large artery atherosclerosis	20 (44.4%)	93 (31.0%)	3.211	0.073	0.180
Small vessel occlusion	12 (26.7%)	166 (55.3%)	12.876	<0.001	0.009
Baseline NIHSS score	11 (6, 17)	7 (4, 12)	−3.211	0.001	0.009
NIHSS score (1 h after IVT)	8 (4, 17)	4 (2, 10)	−3.257	0.001	0.009
NIHSS score (2 h after IVT)	8 (4, 15)	4 (2, 10)	−3.150	0.002	0.014
**Baseline laboratory test**					
WBC, 10^9^/l	6.55 (4.95, 9.11)	7.36 (6.11, 8.84)	−1.852	0.064	0.169
RBC, 10^9^/l	4.36 (3.98, 4.77)	4.69 (4.37, 5.02)	−3.221	0.001	0.009
Hemoglobin, g/l	133 (124, 145)	143 (132, 153)	−3.466	0.001	0.009
Platelet, 10^9^/l	174 (144, 221)	193 (159, 241)	−2.012	0.044	0.141
Neutrophil, 10^9^/l	4.54 (3.05, 6.63)	4.51 (3.53, 6.16)	−0.370	0.711	0.813
Lymphocyte, 10^9^/l	1.45 (1.11, 2.24)	2.05 (1.37, 2.57)	−2.799	0.005	0.029
NLR	2.76 (1.73, 5.02)	2.29 (1.53, 3.82)	−1.466	0.143	0.277
PT, s	11.85 (11.28, 12.65)	11.4 (10.8, 12.0)	−3.137	0.002	0.014
APTT, s	28.91 ± 4.54	28.23 ± 3.86	−1.029	0.304	0.463
Fibrinogen, g/l	3.46 ± 0.80	3.32 ± 0.86	−1.039	0.299	0.463
INR	1.07 (1.03, 1.14)	1.05 (0.99, 1.10)	−2.667	0.008	0.043
Blood glucose, mmol/l	7.91 (6.48, 10.93)	7.13 (6.08, 8.87)	−1.875	0.061	0.169
AKP, u/l	85.0 (69.5, 96.3)	80.5 (67.0, 97.0)	−0.506	0.613	0.772
Albumin, g/l	41.9 (39.0, 45.5)	42.1 (39.2, 44.9)	−0.072	0.942	0.942
Blood urea nitrogen, mmol/l	6.48 (5.63, 7.80)	6.20 (5.00, 7.40)	−1.714	0.086	0.197
Creatinine, μmol/l	75 (67, 88)	73 (62, 87)	−1.040	0.298	0.463
Uric acid, μmol/l	326 (264, 402)	342.0 (280.0, 411.0)	−0.588	0.557	0.754
Potassium, mmol/l	3.77 ± 0.52	3.81 ± 0.45	0.470	0.639	0.772
Sodium, mmol/l	140.6 (138.2, 143.7)	140 (138, 142)	−1.505	0.132	0.273
Chloride, mmol/l	103.6 (101.0, 105.9)	103.8 (101.4, 105.7)	−0.090	0.929	0.942
Calcium, mmol/l	2.27 (2.18, 2.42)	2.29 (2.22, 2.39)	−0.300	0.764	0.815
Phosphorus, mmol/l	1.08 (0.96, 1.20)	1.08 (0.94, 1.19)	−0.482	0.630	0.772
C–reactive protein, mg/l	7.40 (3.47, 19.10)	3.94 (0.82, 8.84)	−2.438	0.015	0.069
**Laboratory test on the second day after IVT**					
Neutrophil after IVT, 10^9^/l	5.52 (4.73, 9.48)	5.79 (4.25, 7.16)	−1.141	0.254	0.428
Lymphocyte after IVT, 10^9^/l	1.19 (0.86, 1.69)	1.36 (0.94, 1.88)	−1.452	0.146	0.277
NLR after IVT	4.61 (2.67, 8.78)	3.83 (2.39, 7.02)	−0.802	0.423	0.630
Potassium after IVT, mmol/l	3.65 (3.33, 3.89)	3.74 (3.58, 3.97)	−2.207	0.027	0.102
Sodium after IVT, mmol/l	139.5 (138.2,141.1)	139.6 (138.0,141.1)	−0.395	0.693	0.810
Chloride after IVT, mmol/l	104.1 (101.6, 105.9)	105.2 (102.7, 107.0)	−1.836	0.066	0.169
Calcium after IVT, mmol/l	2.21 ± 0.13	2.17 ± 0.10	−1.921	0.061	0.169
Phosphorus after IVT, mmol/l	1.01 (0.81, 1.12)	1.01 (0.87, 1.12)	−0.563	0.573	0.754
Tg, mmol/l	0.88 (0.69, 1.25)	1.26 (0.87, 1.83)	−3.666	<0.001	0.009
CHOL, mmol/l	4.32 ± 1.23	4.54 ± 1.04	1.260	0.208	0.370
HDL, mmol/l	1.24 (1.00, 1.48)	1.21 (1.02, 1.39)	−0.313	0.754	0.815
LDL, mmol/l	2.58 (1.97, 3.10)	2.71 (2.20,3.26)	−1.095	0.273	0.448
ApoA−1, g/l	1.15 (0.98, 1.30)	1.10 (0.97, 1.31)	−0.074	0.941	0.942
ApoB, g/l	0.80 (0.60, 0.94)	0.84 (0.70, 1.00)	−1.842	0.065	0.169
Lpa, mg/l	237.5 (130.5, 357.5)	148.0 (69.8, 285.0)	−2.305	0.021	0.084

*PAT, previous antiplatelet therapy; SBP, systolic blood pressure; DBP, diastolic blood pressure; NLR, neutrophil-to-lymphocyte ratio; OTT, onset-to-treatment time; WBC, white blood cell; RBC, red blood cell; AKP, alkaline phosphatase; BUN, blood urea nitrogen; HDL, high-density lipoprotein cholesterol; LDL, low-density lipoprotein cholesterol, Tg, triglycerides; ApoA-1, Apolipoprotein A 1; ApoB, Apolipoprotein B; Lpa, lipoprotein (a); CHOL, cholesterol; Significant difference between the 2 groups (FDR-adjusted P-value < 0.05)*.

### Comparison of the Models for the Prediction of Hemorrhage Transformation in Acute Ischemic Stroke After Intravenous Alteplase

**Figure 3:**
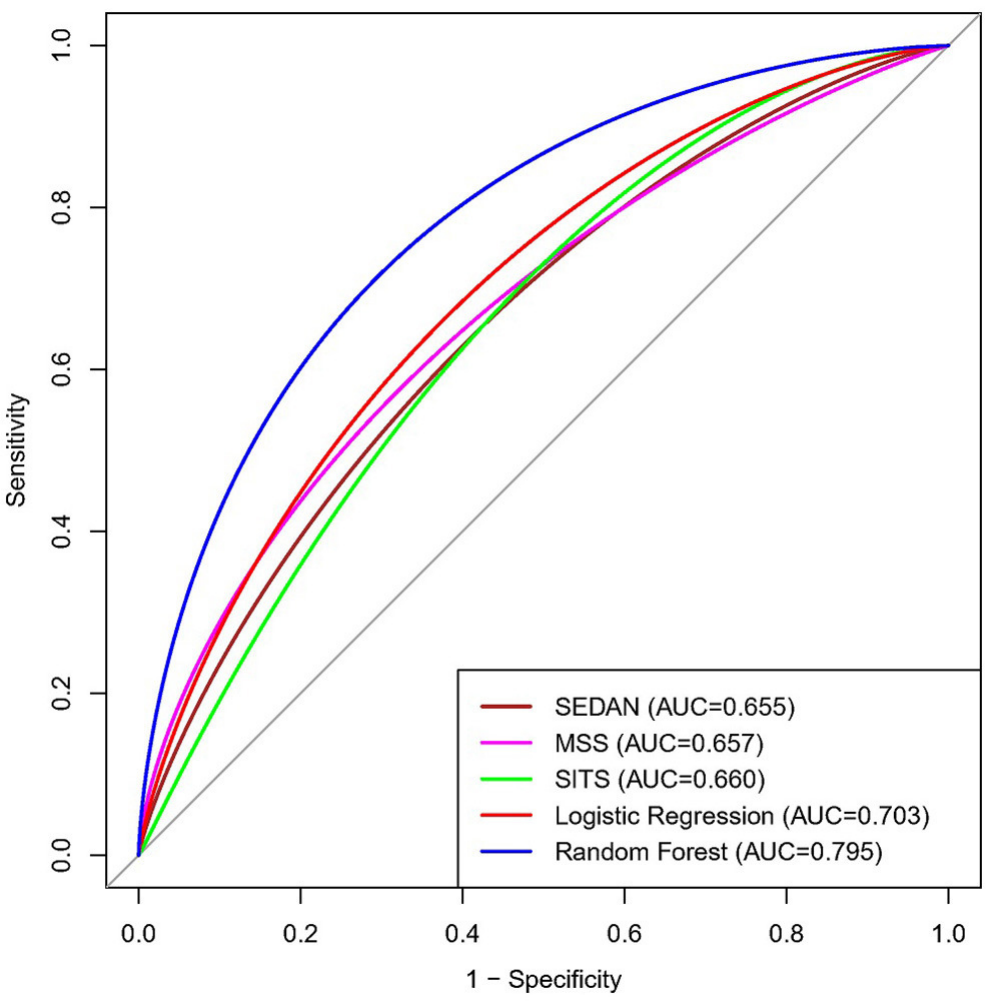
Area under the receiver operating characteristics curves (AUC) for predicting HT in AIS after Intravenous Alteplase. HT, hemorrhage transformation; AIS, acute ischemic stroke.

**Figure 4:**
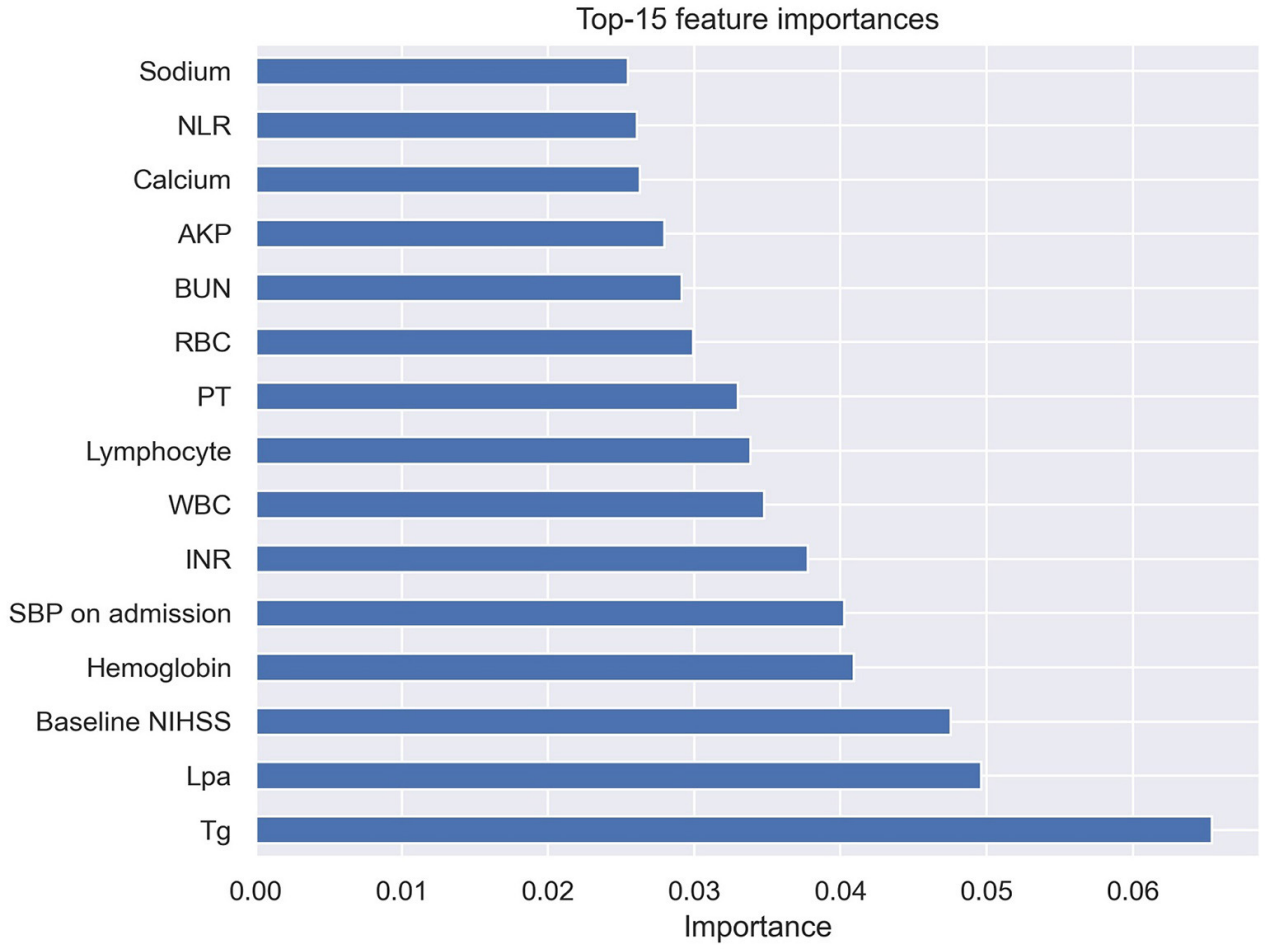
Importance matrix plot of the RF model. Top 15 parameters for predicting HT of AIS after IVT. RF, random forest; HT, hemorrhage transformation; AIS, acute ischemic stroke; IVT, intravenous thrombolysis.

The ROC curve results showed that the AUCs of HT, as predicted by the RF model, LR model, MSS, SITS, and SEDAN scores after IVT in AIS patients were 0.795 (95% CI, 0.647–0.944), 0.703 (95% CI, 0.515–0.892), 0.657 (95% CI, 0.574–0.741), 0.660 (95% CI, 0.580–0.740) and 0.655 (95% CI, 0.571–0.739), respectively. When the optimal cutoff value of the RF model was 0.169, the sensitivity was 66.7%, and the specificity was 80.7%. When the optimal cutoff value of the LR model was 0.140, the sensitivity was 60.0%, and the specificity was 78.0%. The RF model performed significantly better than the other models. The AUCs are presented in Figure 3. The importance matrix plot for the RF method is shown in Figure 4 and reveals that the top 4 parameters with the greatest importance for predicting HT of AIS after IVT in this model with all available parameters were triglyceride, Lpa, the baseline NIHSS and hemoglobin.

### SHAP Value-Based Interpretation of the RF Model

**Figure 5:**
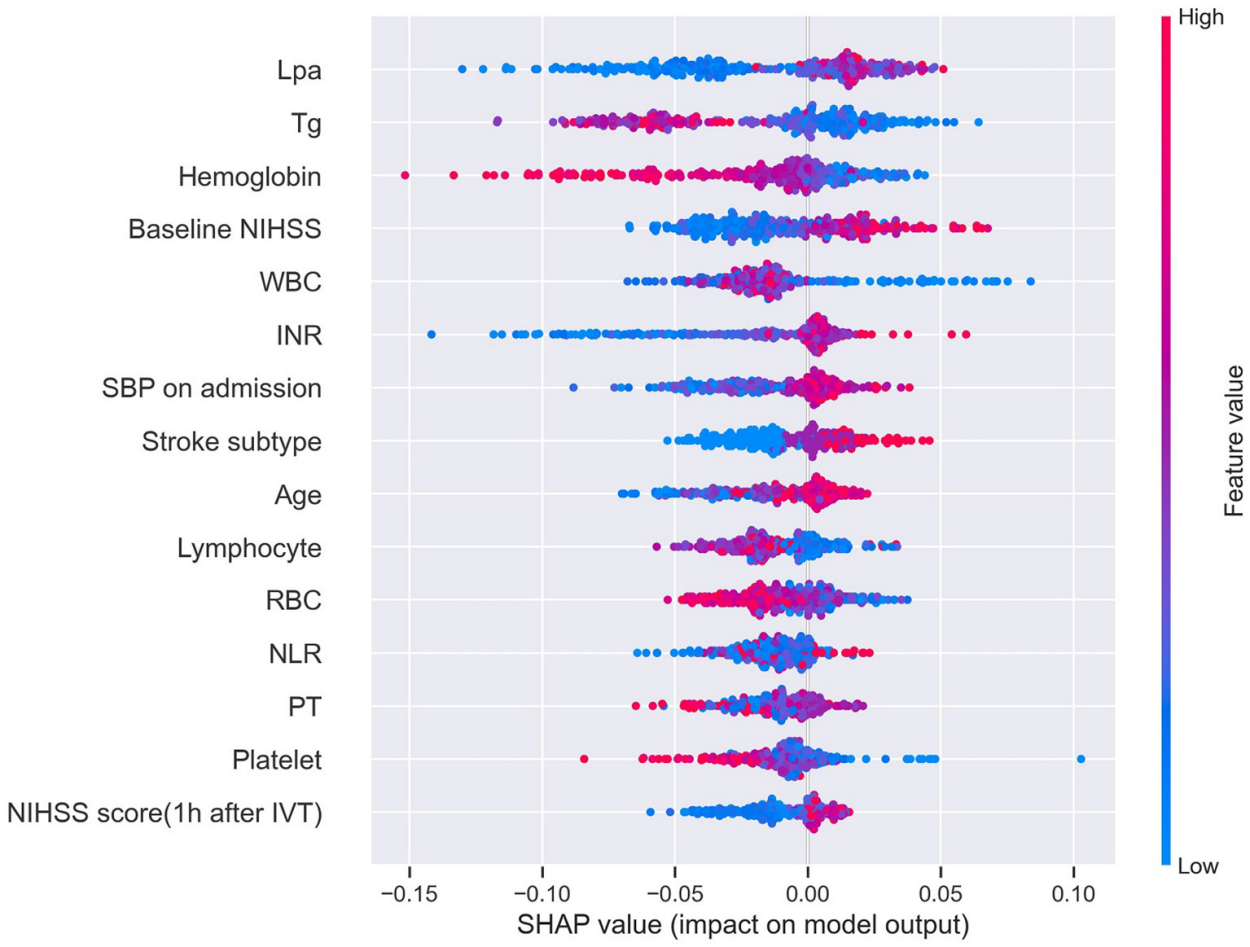
SHAP summary plot of the top 15 features of the RF model. The higher the SHAP value of a feature (*x*-axis), the higher the probability of HT after IVT. Taking the feature Lpa as an example, red points are on the right whereas blue points are on the left. This means prediction scores will be smaller when patients have a low level of Lpa. SHAP, SHapley Additive exPlanation; RF, random forest; HT, hemorrhage transformation; IVT, intravenous thrombolysis; Tg, triglycerides; WBC, white blood cell; RBC, red blood cell; NLR, neutrophil-to-lymphocyte ratio.

**Figure 6:**
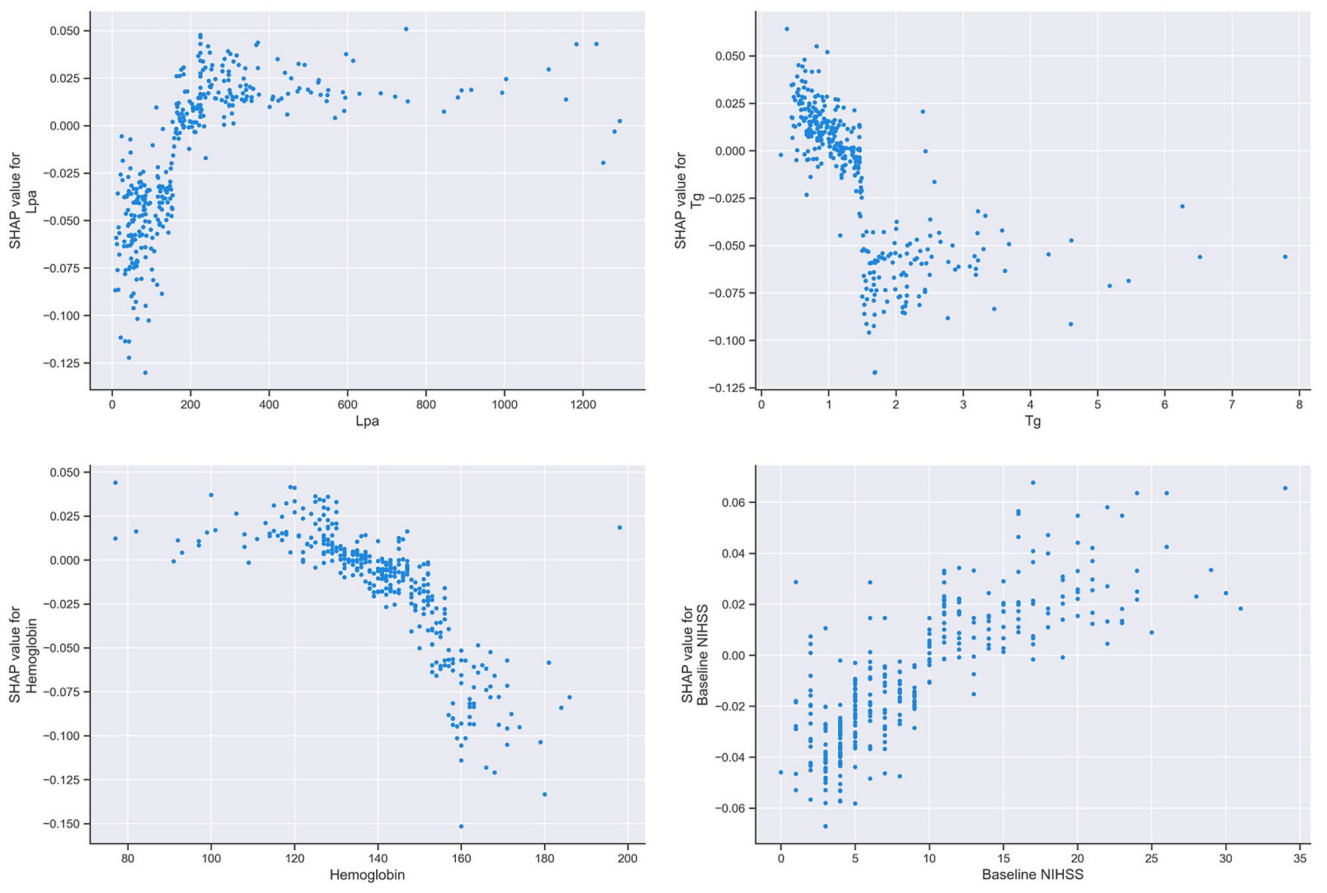
SHAP dependence plot of the RF model. The SHAP dependence plot shows how a single feature affects the output of the RF prediction model. SHAP values for specific features exceed zero, representing an increased risk of HT after IVT. SHAP, SHapley Additive exPlanation; RF, random forest; HT, hemorrhage transformation; IVT, intravenous thrombolysis; Tg, triglycerides; Lpa, lipoprotein(a).

A SHAP summary plot was used to illustrate the positive or negative effects of the features attributed to the RF model (Figure 5). The SHAP dependence plot showed how a single feature of the top 4 factors affected the output of the RF prediction model (Figure 6).

## Discussion

In this retrospective cohort study, we developed and validated machine learning algorithms using 61 features to predict HT after IVT for AIS. The results demonstrated that the use of machine learning models can accurately predict HT. The RF model performed better than the LR model in predicting HT.

Numerous factors affect, with varying significance and mechanisms, the development of HT after IVT in AIS patients. To avoid HT as much as possible, it is important to consider as many influential factors as possible before proceeding with IVT. Unfortunately, the scales that are commonly used in the clinic, such as the MSS score (15), SITS score (16), and SEDAN score (17), are only calculated based on a few important variables. However, machine learning could provide a multi-characteristic analysis in deciphering the factors that are associated with the risk of HT after IVT.

Many risk factors have been confirmed to be associated with HT after IVT. A systematic review and meta-analysis of 55 studies reported that age, severity of the stroke, baseline glucose, use of antiplatelet drugs or statins, leukoaraiosis, early signs of infarction on head CT, and the presence of atrial fibrillation, diabetes, previous ischemic heart disease or cerebral vascular diseases, and congestive heart failure were associated with HT after IVT (10). In addiction, hypertension (23–25) and the NLR (12, 26–28) were also shown to be associated with HT after IVT. The results of the present study confirmed the results of previous reports. Meanwhile, the baseline NIHSS, systolic blood pressure on admission, and the NLR were ranked the top six features in the present study, and the higher their values were, the greater the risk of HT.

The NIHSS score is widely used to assess the clinical stroke severity. It has been suggested that the baseline NIHSS score is an independent risk factor for HT after IVT and that a high NIHSS score is a predictor of HT (25, 29). Moreover, it was reported that 1.6% of IVT patients had fatal HT when their NIHSS score was 5 to 10, while it increased to 6.8% when their NIHSS score was ≥ 22 (9).

The patient's blood pressure (24), especially systolic blood pressure before IVT (23, 25) was also previously shown to be a risk factor for HT. This result was consistent with the present study. This may be because if patients have higher blood pressure, there is more damage to the vascular endothelial cells.

A high NLR was reported to predict HT in AIS patients after IVT in previous studies (12, 26–28). The NLR on admission rather than the NLR post-IVT was an independent risk factor for an increased risk of HT after IVT (28). The results in this study were the same as the results of the above studies. The underlying mechanism of HT remains uncertain. It has been suggested that the NLR is associated with the inflammatory destruction of neutrophils and the protective effect of lymphocytes. Neutrophils plays a role in the disruption of the blood brain barrier (BBB) through inflammation (30, 31) and enhance the expression of matrix metalloproteinase-9 (MMP-9) (32). Rt-PA can not only promote neutrophils to release MMPs but also promote the migration of neutrophils to ischemic tissue (33). Moreover, the stress response leads to an increase in the production of catecholamines in an overactivated sympathetic nervous system, resulting in a decrease in the number and activity of lymphocytes (34).

Our study also obtained some new findings. The factors that were ranked in the top four factors in the present study were triglyceride, Lpa, the baseline NIHSS and hemoglobin, among which, triglyceride, Lpa, and hemoglobin were negelected by commonly used scales in clinic currently.

Several studies have shown that a high level of triglycerides contributes to atherosclerosis (35–37), while a low level is a risk factor for cerebral hemorrhage (38). It was also reported that a low level of triglycerides was a modest risk factor for HT after IVT (11). In the RF model-based machine learning, triglycerides were negatively associated with HT after IVT. This is probably because a high level of triglycerides is correlated with elevations in coagulation factor VII and plasminogen activator inhibitor, and the viscosity of blood and plasma (39).

The results from previous reports on the association between Lpa and stroke are controversial. Several studies have shown that there is no evidence of an association between the Lpa levels and stroke (35, 40). However, a meta-analysis suggested that Lpa was a significant risk factor for ischemic stroke (41). In the present study, we reveal that Lpa as a positive factor that increases HT after IVT, which is a novel finding. Therefore, more detailed studies on the relationship between lipids and the risk of AIS and HT after IVT may be important and informative.

It has been demonstrated that lower hemoglobin levels on admission are associated with hematoma expansion (HE) after intracranial hemorrhage and worse clinical outcomes (42). As indicated by the present study, it was also implicated to be associated with the development of HT after IVT. This may be the result of coagulopathies and prolonged bleeding in anemic patients (43).

There are several limitations in the present study. First, this was a single-center retrospective study, and this study requires validation with larger datasets from other sources. Additionally, the data was too small to further analysis the prediction of SICH. Second, the algorithm was built from the input features, and some hidden relationships may have been ignored because unknown or neglected features were not evaluated by physicians. Third, the patients did not have susceptibility-weighted imaging (SWI), which might have led to an underestimation of HT.

## Conclusion

This study demonstrated that machine learning algorithms, especially the RF model, can improve HT prediction for ischemic stroke patients after intravenous alteplase. It may aid clinicians, as well as patients and families, in the process of decision-making when determining the AIS patient's eligibility for thrombolytic treatment.

## Data Availability Statement

The original contributions presented in the study are included in the article/supplementary material, further inquiries can be directed to the corresponding author/s.

## Ethics Statement

The studies involving human participants were reviewed and approved by the Research Ethics Committee of Affiliated ZhongDa Hospital and the Southeast University. Written informed consent for participation was not required for this study in accordance with the national legislation and the institutional requirements.

## Author Contributions

YX and XL conceived and designed the research. ZZ and AJ provided technical assistance. YX and DW performed the experiments and plotted the figures. YX wrote the manuscript. XL checked the manuscript and made final modifications. All authors have read and approved the submitted version.

## Funding

This study was supported by the Nanjing Medical Science and Technique Development Foundation (No. YKK21217).

## Conflict of Interest

The authors declare that the research was conducted in the absence of any commercial or financial relationships that could be construed as a potential conflict of interest.

## Publisher's Note

All claims expressed in this article are solely those of the authors and do not necessarily represent those of their affiliated organizations, or those of the publisher, the editors and the reviewers. Any product that may be evaluated in this article, or claim that may be made by its manufacturer, is not guaranteed or endorsed by the publisher.

## References

[1] Wang W Jiang B Sun H Ru X Sun D Wang L. Prevalence, incidence, and mortality of stroke in China: results from a Nationwide Population-Based Survey of 480 687 adults. Circulation. (2017) 135:759–71. 10.1161/CIRCULATIONAHA.116.025250 28052979

[2] Ahmed N Wahlgren N Grond M Hennerici M Lees KR Mikulik R. Implementation and outcome of thrombolysis with alteplase 3-4.5 h after an acute stroke: an updated analysis from SITS-ISTR. Lancet Neurol. (2010) 9:866–74. 10.1016/S1474-4422(10)70165-4 20667790

[3] Hacke W Kaste M Bluhmki E Brozman M Davalos A Guidetti D. Thrombolysis with alteplase 3 to 4.5 hours after acute ischemic stroke. N Engl J Med. (2008) 359:1317–29. 10.1056/NEJMoa0804656 18815396

[4] Powers WJ Rabinstein AA Ackerson T Adeoye OM Bambakidis NC Becker K. 2018 Guidelines for the early management of patients with acute ischemic stroke: a guideline for healthcare professionals from the American Heart Association/American Stroke Association. Stroke. (2018) 49:e46–e110. 10.1161/STR.0000000000000158 29367334

[5] Sardar P Chatterjee S Giri J Kundu A Tandar A Sen P. Endovascular therapy for acute ischaemic stroke: a systematic review and meta-analysis of randomized trials. Eur Heart J. (2015) 36:2373–80. 10.1093/eurheartj/ehv270 26071599

[6] Zi W Qiu Z Li F Sang H Wu D Luo W. Effect of endovascular treatment alone vs intravenous alteplase plus endovascular treatment on functional independence in patients with acute ischemic stroke: The DEVT Randomized Clinical Trial. JAMA. (2021) 325:234–43. 10.1001/jama.2020.23523 33464335 PMC7816099

[7] von Kummer R Broderick JP Campbell BC Demchuk A Goyal M Hill MD. The Heidelberg bleeding classification: classification of bleeding events after ischemic stroke and reperfusion therapy. Stroke. (2015) 46:2981–6. 10.1161/STROKEAHA.115.010049 26330447

[8] Lindley RI Wardlaw JM Sandercock PA Rimdusid P Lewis SC Signorini DF. Frequency and risk factors for spontaneous hemorrhagic transformation of cerebral infarction. J Stroke Cerebrovasc Dis. (2004) 13:235–46. 10.1016/j.jstrokecerebrovasdis.2004.03.003 17903981

[9] Emberson J Lees KR Lyden P Blackwell L Albers G Bluhmki E. Effect of treatment delay, age, and stroke severity on the effects of intravenous thrombolysis with alteplase for acute ischaemic stroke: a meta-analysis of individual patient data from randomised trials. Lancet. (2014) 384:1929–35. 10.1016/S0140-6736(14)60584-5 25106063 PMC4441266

[10] Whiteley WN Slot KB Fernandes P Sandercock P Wardlaw J. Risk factors for intracranial hemorrhage in acute ischemic stroke patients treated with recombinant tissue plasminogen activator: a systematic review and meta-analysis of 55 studies. Stroke. (2012) 43:2904–9. 10.1161/STROKEAHA.112.665331 22996959

[11] Messe SR Pervez MA Smith EE Siddique KA Hellkamp AS Saver JL. Lipid profile, lipid-lowering medications, and intracerebral hemorrhage after tPA in get with the guidelines-stroke. Stroke. (2013) 44:1354–9. 10.1161/STROKEAHA.111.671966 23493734

[12] Liu YL Lu JK Yin HP Xia PS Qiu DH Liang MQ. High neutrophil-to-lymphocyte ratio predicts hemorrhagic transformation in acute ischemic stroke patients treated with intravenous thrombolysis. Int J Hypertens. (2020) 2020:5980261. 10.1155/2020/5980261 32181011 PMC7064843

[13] Liu L Teng J Ma M Guo L Yang L Gao J. Serum homocysteine level is an independent predictor for hemorrhagic transformation within 24 h of intravenous thrombolysis in acute ischemic stroke. J Clin Neurosci. (2020) 82:13–9. 10.1016/j.jocn.2020.10.021 33317721

[14] Lou M Safdar A Mehdiratta M Kumar S Schlaug G Caplan L. The HAT Score: a simple grading scale for predicting hemorrhage after thrombolysis. Neurology. (2008) 71:1417–23. 10.1212/01.wnl.0000330297.58334.dd 18955684 PMC2676961

[15] Cucchiara B Tanne D Levine SR Demchuk AM Kasner S. A risk score to predict intracranial hemorrhage after recombinant tissue plasminogen activator for acute ischemic stroke. J Stroke Cerebrovasc Dis. (2008) 17:331–3. 10.1016/j.jstrokecerebrovasdis.2008.03.012 18984422

[16] Mazya M Egido JA Ford GA Lees KR Mikulik R Toni D. Predicting the risk of symptomatic intracerebral hemorrhage in ischemic stroke treated with intravenous alteplase safe implementation of treatments in stroke (SITS) symptomatic intracerebral hemorrhage risk score. Stroke. (2012) 43:1524–31. 10.1161/STROKEAHA.111.644815 22442178

[17] Strbian D Engelter S Michel P Meretoja A Sekoranja L Ahlhelm FJ. Symptomatic intracranial hemorrhage after stroke thrombolysis: the SEDAN score. Ann Neurol. (2012) 71:634–41. 10.1002/ana.23546 22522478

[18] Menon BK Saver JL Prabhakaran S Reeves M Liang L Olson DM. Risk score for intracranial hemorrhage in patients with acute ischemic stroke treated with intravenous tissue-type plasminogen activator. Stroke. (2012) 43:2293–9. 10.1161/STROKEAHA.112.660415 22811458

[19] Cruz JA Wishart DS. Applications of machine learning in cancer prediction and prognosis. Cancer Inform. (2007) 2:59–77. 10.1177/117693510600200030 19458758 PMC2675494

[20] Heo J Yoon JG Park H Kim YD Nam HS Heo JH. Machine learning-based model for prediction of outcomes in acute stroke. Stroke. (2019) 50:1263–5. 10.1161/STROKEAHA.118.024293 30890116

[21] Brugnara G Neuberger U Mahmutoglu MA Foltyn M Herweh C Nagel S. Multimodal predictive modeling of endovascular treatment outcome for acute ischemic stroke using machine-learning. Stroke. (2020) 51:3541–51. 10.1161/STROKEAHA.120.030287 33040701

[22] Lundberg SM Erion G Chen H DeGrave A Prutkin JM Nair B. From local explanations to global understanding with explainable AI for trees. Nat Mach Intell. (2020) 2:56–67. 10.1038/s42256-019-0138-9 32607472 PMC7326367

[23] Liu K Yan S Zhang S Guo Y Lou M. Systolic blood pressure variability is associated with severe hemorrhagic transformation in the early stage after thrombolysis. Transl Stroke Res. (2016) 7:186–91. 10.1007/s12975-016-0458-6 26892891

[24] Tanne D Kasner SE Demchuk AM Koren-Morag N Hanson S Grond M. Markers of increased risk of intracerebral hemorrhage after intravenous recombinant tissue plasminogen activator therapy for acute ischemic stroke in clinical practice: the Multicenter rt-PA Stroke Survey. Circulation. (2002) 105:1679–85. 10.1161/01.CIR.0000012747.53592.6A 11940547

[25] Xu XH Li CS Wan T Gu XB Zhu WX Hao JJ. Risk factors for hemorrhagic transformation after intravenous thrombolysis in acute cerebral infarction: a retrospective single-center study. World Neurosurg. (2017) 101:155–60. 10.1016/j.wneu.2017.01.091 28185970

[26] Guo Z Yu S Xiao L Chen X Ye R Zheng P. Dynamic change of neutrophil to lymphocyte ratio and hemorrhagic transformation after thrombolysis in stroke. J Neuroinflamm. (2016) 13:199. 10.1186/s12974-016-0680-x 27561990 PMC5000487

[27] Song Q Li Y Wang Y Wei C Liu J Liu M. Increased neutrophil-to-lymphocyte ratios are associated with greater risk of hemorrhagic transformation in patients with acute ischemic stroke. Curr Neurovasc Res. (2018) 15:326–35. 10.2174/1567202616666181204122457 30514190

[28] Wang C Zhang Q Ji M Mang J Xu Z. Prognostic value of the neutrophil-to-lymphocyte ratio in acute ischemic stroke patients treated with intravenous thrombolysis: a systematic review and meta-analysis. BMC Neurol. (2021) 21:191. 10.1186/s12883-021-02222-8 33975565 PMC8111766

[29] Kidwell CS Saver JL Carneado J Sayre J Starkman S Duckwiler G. Predictors of hemorrhagic transformation in patients receiving intra-arterial thrombolysis. Stroke. (2002) 33:717–24. 10.1161/hs0302.104110 11872894

[30] Jickling GC Liu D Ander BP Stamova B Zhan X Sharp FR. Targeting neutrophils in ischemic stroke: translational insights from experimental studies. J Cereb Blood Flow Metab. (2015) 35:888–901. 10.1038/jcbfm.2015.45 25806703 PMC4640255

[31] Jin R Liu L Zhang S Nanda A Li G. Role of inflammation and its mediators in acute ischemic stroke. J Cardiovasc Transl Res. (2013) 6:834–51. 10.1007/s12265-013-9508-6 24006091 PMC3829610

[32] Inzitari D Giusti B Nencini P Gori AM Nesi M Palumbo V. MMP9 variation after thrombolysis is associated with hemorrhagic transformation of lesion and death. Stroke. (2013) 44:2901–3. 10.1161/STROKEAHA.113.002274 23908067

[33] Ma G Pan Z Kong L Du G. Neuroinflammation in hemorrhagic transformation after tissue plasminogen activator thrombolysis: Potential mechanisms, targets, therapeutic drugs and biomarkers. Int Immunopharmacol. (2021) 90:107216. 10.1016/j.intimp.2020.107216 33296780

[34] Liu DD Chu SF Chen C Yang PF Chen NH He X. Research progress in stroke-induced immunodepression syndrome (SIDS) and stroke-associated pneumonia (SAP). Neurochem Int. (2018) 114:42–54. 10.1016/j.neuint.2018.01.002 29317279

[35] Hachinski V Graffagnino C Beaudry M Bernier G Buck C Donner A. Lipids and stroke: a paradox resolved. Arch Neurol. (1996) 53:303–8. 10.1001/archneur.1996.00550040031011 8929151

[36] Nordestgaard BG. Triglyceride-rich lipoproteins and atherosclerotic cardiovascular disease: new insights from epidemiology, genetics, and biology. Circ Res. (2016) 118:547–63. 10.1161/CIRCRESAHA.115.306249 26892957

[37] Sascau R Clement A Radu R Prisacariu C Statescu C. Triglyceride-rich lipoproteins and their remnants as silent promoters of atherosclerotic cardiovascular disease and other metabolic disorders: a review. Nutrients. (2021) 13:1774. 10.3390/nu13061774 34067469 PMC8224751

[38] An SJ Kim TJ Yoon BW. Epidemiology, risk factors, and clinical features of intracerebral hemorrhage: an update. J Stroke. (2017) 19:3–10. 10.5853/jos.2016.00864 28178408 PMC5307940

[39] Rosenson RS Lowe GD. Effects of lipids and lipoproteins on thrombosis and rheology. Atherosclerosis. (1998) 140:271–80. 10.1016/S0021-9150(98)00144-0 9862270

[40] Alfthan G Pekkanen J Jauhiainen M Pitkaniemi J Karvonen M Tuomilehto J. Relation of serum homocysteine and lipoprotein (A) concentrations to atherosclerotic disease in a prospective finnish population-based study. Atherosclerosis. (1994) 106:9–19. 10.1016/0021-9150(94)90078-7 8018111

[41] Nave AH Lange KS Leonards CO Siegerink B Doehner W Landmesser U. Lipoprotein (a) as a risk factor for ischemic stroke: a meta-analysis. Atherosclerosis. (2015) 242:496–503. 10.1016/j.atherosclerosis.2015.08.021 26298741

[42] Roh DJ Albers DJ Magid-Bernstein J Doyle K Hod E Eisenberger A. Low hemoglobin and hematoma expansion after intracerebral hemorrhage. Neurology. (2019) 93:e372–80. 10.1212/WNL.0000000000007820 31209179 PMC6669932

[43] Livio M Gotti E Marchesi D Mecca G Remuzzi G de Gaetano G. Uraemic bleeding: role of anaemia and beneficial effect of red cell transfusions. Lancet. (1982) 2:1013–5. 10.1016/S0140-6736(82)90050-2 6127502

